# Lead and cadmium levels in blood among painters and battery workers: A case control study

**DOI:** 10.6026/973206300223369

**Published:** 2026-06-30

**Authors:** Durgesh Kumar, Mohammad Kaleem Ahmad, Anveshika Manoj, Gautam Prasad, Ravindra Kumar Garg, Abbas Ali Mahdi

**Affiliations:** 1 Department of Biochemistry, King George's Medical University, Lucknow, 226003, India; 2 Department of Neurology, King George's Medical University, Lucknow, 226003, India

**Keywords:** Occupational exposure, metal toxicity, oxidative stress, total antioxidant capacity

## Abstract

Occupational exposure to heavy metals like lead and cadmium remains a significant health concern due to poor workplace awareness and unsafe practices. Therefore, it is of interest to evaluated blood levels of lead, cadmium and oxidative stress markers in occupationally exposed workers (painters and battery workers) compared to controls. Analysis using ICP-MS revealed significantly elevated metal levels in exposed workers, along with decreased antioxidant enzyme activities and increased lipid peroxidation (p < 0.001). Total antioxidant capacity was also significantly reduced, indicating impaired defense against oxidative stress in exposed individuals. Thus, we report the mechanistic link between heavy metal exposure and oxidative imbalance, emphasizing the need for improved occupational safety and preventive strategies.

## Background:

Occupational exposure to heavy metals such as lead and cadmium continues to be a significant public health concern due to their widespread presence in the environment and extensive industrial use [1]. These metals are introduced into the environment through both natural processes and anthropogenic activities, leading to persistent exposure risks, particularly among industrial workers [2]. Lead has been utilized for thousands of years owing to its favorable physical and chemical properties, including corrosion resistance, malleability and low melting point, which make it suitable for use in industries such as painting, battery manufacturing, plumbing and ceramics [3]. Similarly, cadmium, a non-essential heavy metal, has gained prominence in the last century due to its application in rechargeable batteries, pigments, electroplating and plastics. Despite regulatory measures, exposure to these metals remains prevalent, especially in developing countries like India, where occupational safety practices may be inadequate [4]. Both lead and cadmium are known to exert toxic effects on multiple organ systems, contributing to the development of various diseases. One of the key mechanisms underlying their toxicity is the induction of oxidative stress [5]. Oxidative stress occurs due to an imbalance between the generations of reactive oxygen species (ROS) and the body's antioxidant defense system. These metals promote the formation of free radicals, which can damage cellular components such as lipids, proteins and DNA, ultimately impairing cellular function and integrity [6]. Lead, in particular, has a strong affinity for sulfhydryl groups, which disrupts calcium homeostasis and depletes antioxidant reserves in the body. Cadmium exposure has been associated with carcinogenic effects, affecting organs such as the lungs, kidneys, prostate and liver and is classified as a human carcinogen by international health agencies [7]. Common routes of exposure include inhalation, ingestion of contaminated food or water and lifestyle factors such as smoking. Occupational groups such as painters and battery workers are especially vulnerable to chronic exposure due to direct contact with these metals in their work environment [8]. In many cases, lack of awareness and unsafe practices such as eating, drinking, or smoking at the workplace further increase the risk of absorption and toxicity. Although guidelines have been established to define permissible levels of lead and cadmium in the blood, studies indicate that even low levels of exposure can have significant biological effects, particularly by altering oxidative stress markers [9]. In this context, the present study was designed to evaluate the levels of lead and cadmium in the blood of occupationally exposed workers, specifically painters and battery workers and to assess their relationship with oxidative stress markers [10]. Both enzymatic antioxidants, including catalase, superoxide dismutase, glutathione peroxidase and glutathione reductase and non-enzymatic indicators such as total antioxidant capacity and malondialdehyde levels were considered. By comparing these parameters with those of non-exposed controls, the study aimed to identify the extent of oxidative imbalance associated with heavy metal exposure [11]. Therefore, it is of interest to assess the relationship between occupational exposure to heavy metals and oxidative stress, which may contribute to the development of preventive strategies and improved workplace safety measures.

## Materials and Methods:

This case-control study included a total of 260 participants divided into two groups: 130 occupationally exposed workers (painters, n = 60; battery workers, n = 70) aged 20-60 years with at least two years of exposure to lead and 130 age-matched healthy controls. Ethical approval was obtained from the Institutional Ethics Committee of King George's Medical University, Lucknow (Ref. Code: 113th ECM llB-Ph.D/P6) and written informed consent was secured from all participants. A detailed questionnaire was used to collect demographic, occupational and lifestyle data, including dietary habits, smoking, alcohol consumption and medical history. Individuals with chronic illnesses such as diabetes, cardiovascular diseases, cancer, immunological disorders, or recent infections were excluded to minimize bias. Venous blood (5 ml) was collected from each subject in EDTA vials. Whole blood was used for estimation of lead and cadmium levels, while plasma and lysate were separated for oxidative stress analysis and stored at -20°C until further use. Plasma was obtained by centrifugation at 2200-2500 rpm for 10-15 minutes and carefully transferred into labeled sterile tubes. For lysate preparation, the red blood cell pellet was washed three times with normal saline at 10,000 rpm and 4°C, followed by treatment with chilled distilled water and centrifugation. The supernatant obtained was stored under appropriate conditions for further biochemical analysis. Blood lead and cadmium levels were estimated using Inductively Coupled Plasma-Mass Spectrometry (ICP-MS, NexIon 2000) following microwave digestion. Calibration standards were prepared from stock solutions and diluted appropriately. For digestion, 0.5 ml of blood sample was treated with nitric acid and subjected to controlled microwave digestion. The digested samples were diluted to a final volume of 25 ml using triple-distilled water and analyzed, with results expressed in µg/dl. Oxidative stress markers were evaluated by measuring enzymatic antioxidants, including catalase (Aebi *et al.*, 1974), superoxide dismutase (McCord and Fridovich, 1969), glutathione reductase (Hazelton and Lang) and glutathione peroxidase (Paglia and Valentine, 1967). Lipid peroxidation was assessed by estimating malondialdehyde (MDA) levels using the thiobarbituric acid reactive substances (TBARS) method. Total antioxidant capacity (T-AOC) was determined using a colorimetric assay kit as per the manufacturer's protocol. Statistical analysis was performed using IBM SPSS version 23. Data normality was assessed using the Kolmogorov-Smirnov test. Comparative analysis between groups was conducted using one-way ANOVA followed by Tukey's post hoc test, while unpaired t-tests were used for comparison between two independent groups. The chi-square test was applied for categorical variables and Pearson's correlation analysis was used to determine relationships between variables. A p-value of <0.05 was considered statistically significant.

## Results:

The demographic characteristics of occupationally exposed workers (painters and battery workers) and control subjects were summarized by the age, gender, use of protective measures, smoking habits, dietary habits, alcohol consumption and previous health history. Age distributions were similar among the groups with no significant differences. Gender distribution showed notably significant differences across the group and females only present in the control and battery workers groups but absent in painters. The use of protective measures showed a significant difference. Regarding smoking habits, there were significant differences in groups. Dietary habits did not significantly differ among the groups. Alcohol consumption showed significant differences among all groups. Based on previous health history, it determined that the painters and battery workers were not suffering from any chronic illnesses represented in Table 1. Results of the study showed that blood lead levels in the painters and battery workers were significantly high (p<0.001) as compared to controls. The mean lead levels found in painters, battery workers and controls were 41.37 ± 20.71 µg/dl, 56.25 ± 17.66 µg/dl and 6.55 ± 2.02 µg/dl, respectively. Cadmium level in painters and battery workers as compared to control were significantly increased (p<0.001). The cadmium level found in painters, battery workers and controls were 0.051 ± 0.034 µg/dl, 0.075 ± 0.047 µg/dl, 0.034 ± 0.039 µg/dl, respectively. To assess the activity of oxidative stress markers caused by exposure to lead typically enzymatic antioxidants, we measured 4 parameters like catalase, superoxide dismutase, glutathione reductase and glutathione peroxidase. Results of the enzymatic antioxidant level revealed that the catalase activity in painters and battery workers was 113.76 ± 15.19 U/ml and 109.91 ± 13.87 U/ml, respectively which was found significantly decreased when compared with controls 134.65 ± 34.72 U/ml. Similarly, superoxide dismutase activity in painters and battery workers was found to be 0.827 ± 0.335 U/ml, 0.700 ± 0.362 U/ml, respectively, which was found to be significantly decreased compared with controls 0.988 ± 0.323 U/ml. Likewise, the activity of glutathione reductase also found significantly decreased in painters and battery workers 187.07 ± 27.48 mmole/ml, 167.77 ± 28.40 mmole/ml, respectively, compared to control 225.96 ± 62.34 mmole/ml. Moreover, glutathione peroxidase activity in painters and battery workers was found 212.58 ± 76.30 mmole/ml and 181.04 ± 84.53 mmole/ml, respectively, which is significantly decreased, compared to control 353.88 ± 175.70 mmole/ml. On the contrary the level of lipid peroxidation product as malondialdehyde in painters and battery workers 11.11 ± 4.25 nmol/ml and 12.71 ± 5.79 nmol/ml, respectively, which was significantly high when compared with controls 3.01 ± 2.11 nmol/ml. Additionally, the activity of total antioxidant capacity was found significantly decreased in painters and battery workers 6.312 ± 4.101 U/µl and 6.170 ± 2.987 U/µl, respectively, when compared with controls 8.658 ± 5.899 U/µl. Among the variables catalase, malondialdehyde, superoxide dismutase, T-AOC, cadmium and lead were found to be positively correlated with age. Out of these variables T-AOC and cadmium levels were significantly (p < 0.05) correlated with age. Moreover, glutathione reductase, malondialdehyde, T-AOC, cadmium and lead were positively correlated with duration of occupation shown in Table 2. The association between male and female exposed to lead and cadmium in relation to oxidative stress markers revealed that lead exposed males were positively correlated with glutathione reductase, malondialdehyde and superoxide dismutase. However, only glutathione reductase and T-AOC were found positively correlated with cadmium exposure. Lead exposed females were positively correlated with malondialdehyde. Furthermore, cadmium exposed females were positively correlated with catalase, glutathione reductase, malondialdehyde and superoxide dismutase represented in Table 3. Pearson's correlation coefficients amongst oxidative stress parameters with lead and cadmium exposure in painters and battery workers are summarized in Table 4. Lead exposed painters were positively correlated with the level of malondialdehyde, T-AOC and the activity of superoxide dismutase while the cadmium exposed painters were positively correlated with catalase, glutathione reductase, superoxide dismutase activity and T-AOC level. However, lead exposed battery workers were positively associated with the activity of glutathione reductase and malondialdehyde. Furthermore, cadmium exposed battery workers were positively correlated with the activity of glutathione peroxidase and glutathione reductase.

## Discussion:

Present study demonstrated that heavy metal exposure was significantly different in painters, battery workers and controls group. Lead levels were substantially higher in painters 41.37 ± 20.71 µg/dl and 56.25 ± 17.66 µg/dl in battery workers when compared with controls 6.55 ± 2.02 µg/dl. The results of the present study are consistent with our previous report; Khan *et al.* in which we reported that painters had higher blood lead levels than controls 21.56 ± 6.43 µg/dl and 2.84 ± 0.96 µg/dl respectively [12]. Goyal *et al.* [1] reported the mean blood lead level of those employed in the welding and metal crafts sectors as 7.97 ± 1.92 µg/dl which is still significantly high when compared to those who were not exposed. Another study by Himani *et al.* reported blood lead levels as 39.5 ± 31.9 µg/dl in battery workers in the Delhi-NCR region [3]. Similarly, higher blood lead levels 25.26 ± 2.121 µg/dl were reported by Singamsetty and Gollapalli in Andhra Pradesh battery workers [13]. Likewise, Kshirsagar *et al.* found that battery workers in Maharashtra had a mean blood lead level of 33.50 ± 12.90 µg/dl [10]. The majority of lead is transported by erythrocytes after being absorbed into the bloodstream. The lead is freely diffusible in plasma component, which is widely dispersed across several tissues, with notable quantities found in bone, teeth, liver, lungs, kidneys, brain and spleen due to slow elimination rate it can accumulate in these organs and eventually cause multisystem toxicity, even at extremely low exposure levels [14]. We also found that the painters and battery workers had higher blood cadmium levels 0.051 ± 0.034 µg/dl and 0.075 ± 0.047 µg/dl, respectively when compared with controls 0.034 ± 0.039 µg/dl. A previous study found that workers at seven smelting plants in central and southwest China had blood cadmium levels of 3.61 ± 0.84 µg/l, which was above the permissible threshold. A recent study found that welders had higher blood cadmium levels (2.93 ± 0.16 µg/l) than other worker groups, possibly due to exposure to welding fumes [15]. It was reported that, cadmium gets absorbed in the liver and stimulates the production of metallothionein. Metallothionein plays a significant function in the retention of cadmium in many tissues. Cadmium binds to metallothionein and is released into the bloodstream, where it can cause deleterious effects resulting in inflammation, fibrosis, mitochondrial damage and cell death [16]. Moreover, present study also revealed that the oxidative stress markers such as catalase, glutathione peroxidase, glutathione reductase, superoxide dismutase and the total antioxidant capacity levels were significantly decreased (p<0.001) in painters and battery workers as compared to controls. Furthermore, lipid peroxidation product, malondialdehyde, was found to be significantly increased (p<0.001) in painters and battery workers when compared with controls. Previous study reported battery manufacturing workers had significantly higher levels of serum LP (p < 0.001, 96.86%) and lower levels of antioxidants such as SOD and CAT (p < 0.001, -26.32%, -51.57%) as compared to control subjects [17]. Another study performed by Hernández-Franco *et al.* (2022) [18] found increased levels of lipid peroxides in cases as compared to controls. It has been reported that the hypertension group had significantly higher blood MDA levels (9.64 µmol/L) compared to the prehypertensive (8.02 µmol/L) and normotensive (8.23 µmol/L) groups. There were not any significant differences in the means of CAT, SOD, GPx and GSH between the three groups [19]. It is suggested that high levels of lead and cadmium may cause generation of reactive oxygen species (ROS) resulting in oxidative stress. The molecular factors behind lead induced oxidative stress include the production of free radicals through the inhibition of heme biosynthesis pathway enzymes, the augmentation of cell membrane peroxidation susceptibility and depletion of the activity of antioxidant enzymes [20]. Oxidative stress impaired membrane lipids, proteins and nucleic acids may damage the tissues, cells and nearly all the body's organs leading to hepatotoxicity, cardiotoxicity, neurotoxicity and nephrotoxicity [21, 22]. Additionally, we observed the association between oxidative stress markers and heavy metals exposure with age and duration of occupation, correlation of oxidative stress markers with lead and cadmium exposure in male versus female and the correlation coefficient among oxidative stress markers with lead and cadmium exposure in painters and battery workers. Out of these variables T-AOC and cadmium level was significantly (p < 0.05) correlated with age in painters and battery workers. Overall, these results indicate significant biochemical alterations and increased heavy metal exposure in painters and battery workers as compared to the controls emphasizing the occupational hazards associated with these professions.

## Conclusion:

Painters and battery workers showed significantly higher blood lead and cadmium levels compared to controls, along with altered oxidative stress markers. Occupational exposure duration and advancing age were associated with increased heavy metal burden and oxidative stress alterations. Improved workplace safety measures, protective equipment and occupational health education are essential to reduce heavy metal exposure among industrial workers.

## Funding:

This work was supported by the Council of Scientific & Industrial Research (Grant No. 09/910(0015)/2019-EMR-I), New Delhi, India.

## Human ethics and consent to participate:

This research study was approved by the Institutional Ethics Committee, King George's Medical University, Lucknow, India, Ref. Code: 113th ECM llB-Ph.D/P6.

## Declaration of competing interest:

The authors affirm that they have no known conflicting financial interests or personal relationships that could have seemed to influence the work stated in this paper.

## Author contributions:

Durgesh Kumar: Data collection, collecting specimens, laboratory analysis, data management analysis and interpretation, manuscript writing. Mohammad Kaleem Ahmad: Supervision of laboratory analysis, drafting the article and critical revision of the article. Anveshika Manoj: Collecting specimens, laboratory analysis, data compilation. Gautam Prasad: Collecting specimens, data collection, laboratory analysis. Ravindra Kumar Garg: Drafting the article and critical revision of the article. Abbas Ali Mahdi: Conception or design of the work, supervision recruitment of subjects, methodology, overall supervision.

## Figures and Tables

**Table 1:** Demographic characteristics of the study subjects

**Demographic Variable**	**Group**	**Control n (%)**	**Painters n (%)**	**Battery Workers n (%)**	**^2^**	**p-value**
Age	20-30 years	27 (20.8)	15 (25.0)	15 (21.4)	0.98	0.987
	30-40 years	43 (33.1)	17 (28.3)	22 (31.4)		
	40-50 years	36 (27.7)	17 (28.3)	18 (25.7)		
	50-60 years	24 (18.5)	11 (18.3)	15 (21.4)		
Gender	Male	98 (75.4)	60 (100.0)	44 (62.9)	26.52	<0.001
	Female	32 (24.6)	0 (0.0)	26 (37.1)		
Use of Protective Measures	No	25 (19.2)	55 (91.7)	62 (88.6)	131.5	<0.001
	Yes	105 (80.8)	5 (8.3)	8 (11.4)		
Smoking Habit	Non-Smoker	110 (84.6)	20 (33.3)	9 (12.9)	106.9	<0.001
	Smoker	20 (15.4)	40 (66.7)	61 (87.1)		
Dietary Habit	Vegetarian	87 (66.9)	33 (55.0)	41 (58.6)	2.93	0.231
	Non-Vegetarian	43 (33.1)	27 (45.0)	29 (41.4)		
Alcohol	Non-Alcoholic	116 (89.2)	31 (51.7)	23 (32.9)	70.37	<0.001
	Alcoholic	14 (10.8)	29 (48.3)	47 (67.1)		
Abbreviation: ^2^:
Chi-square test; p<0.001,
statistically significant.

**Table 2:** Correlation of oxidative stress markers and heavy metals exposure with age and duration of occupation

**Variables**	**Age r-value**	**Age p-value**	**Duration r-value**	**Duration p-value**
Catalase (U/ml)	0.072	0.249	-0.159	0.07
Glutathione Peroxidase (mmole/ml)	-0.033	0.592	-0.121	0.169
Glutathione Reductase (mmole/ml)	-0.085	0.173	0.031	0.723
Malondialdehyde (nmol/ml)	0.079	0.203	0.13	0.139
S.O.D. (U/ml)	-0.016	0.793	-0.077	0.386
T-AOC (U/µl)	0.276	<0.001	0.134	0.129
Cadmium (µg/dl)	0.163	0.008	0.096	0.275
Lead (µg/dl)	0.059	0.341	0.047	0.598
Abbreviation:
S.O.D.: Superoxide Dismutase,
T-AOC: Total antioxidant capacity;
r denotes Pearson's correlation;
p, 2-tailed test.
p<0.05 shows statistically significant

**Table 3:** Correlation of oxidative stress markers with lead and cadmium exposure in male vs female

**Variables**	**Male Pb (r)**	**Male Cd (r)**	**Female Pb (r)**	**Female Cd (r)**
Catalase (U/ml)	-0.281	-0.338	-0.058	0.114
Glutathione Peroxidase (mmole/ml)	-0.329	-0.113	-0.171	-0.105
Glutathione Reductase (mmole/ml)	0.104	0.112	-0.098	0.05
Malondialdehyde (nmol/ml)	0.146	-0.07	0.21	0.033
S.O.D. (U/ml)	0.025	-0.14	-0.198	0.037
T-AOC (U/µl)	-0.111	0.13	-0.002	-0.24
Abbreviation:
Pb: Lead, Cd: Cadmium,
S.O.D.: Superoxide Dismutase,
T-AOC: Total antioxidant capacity;
r denotes Pearson's correlation.

**Table 4:** Pearson's correlation coefficient among oxidative stress markers with lead and cadmium exposure in painters and battery workers

**Variables**	**Painters Pb (r)**	**Painters Cd (r)**	**Battery Pb (r)**	**Battery Cd (r)**
Catalase (U/ml)	-0.016	0.09	-0.203	-0.242
Glutathione Peroxidase (mmole/ml)	-0.178	-0.145	-0.236	0.018
Glutathione Reductase (mmole/ml)	-0.031	0.031	0.047	0.082
Malondialdehyde (nmol/ml)	0.138	-0.003	0.142	-0.015
S.O.D. (U/ml)	0.1	0.061	-0.036	-0.089
T-AOC (U/µl)	0.252	0.183	-0.064	-0.074
Abbreviation: Pb: Lead, Cd: Cadmium,
S.O.D.: Superoxide Dismutase,
T-AOC: Total antioxidant capacity;
r denotes Pearson's correlation.
